# Paring of Skin for Superficially Lodged Foreign Body Removal

**DOI:** 10.7759/cureus.42396

**Published:** 2023-07-24

**Authors:** Kendahl L Oberdorfer, Mehdi Farshchian, Meena Moossavi

**Affiliations:** 1 Dermatology, Wayne State University School of Medicine, Detroit, USA; 2 Dermatology, David Geffen School of Medicine, University of California Los Angeles, Los Angeles, USA; 3 Dermatology, John D. Dingell Veterans Affairs Hospital, Detroit, USA

**Keywords:** splinter, paring, tweezer, medical dermatology, foreign body retrieval

## Abstract

Small foreign bodies superficially embedded in the acral skin can be difficult to remove. Typical treatment includes using forceps and pressure to attempt removal, which is painful and not always successful. Here we present a patient with a prolonged presentation of a superficially embedded foreign body on his finger. The method for extracting the foreign body was successful and painless through the paring of the skin to gently remove the object. This method decreases pain and swelling, making it a more efficient way of extracting small foreign bodies that are lodged in the superficial skin layer. This case report focuses on more efficient and painless removal of superficially lodged foreign body removal in the clinical office setting.

## Introduction

For removing foreign bodies in the skin, forceps hurt and do not always work. Foreign bodies are small objects that enter through the epidermis. The foreign body then travels to the dermis, causing pain and inflammation due to nerve injury and foreign body attack. Examples include splinters, thorns, and cactus spines known as “glochids.” Patients may delay seeking care due to pain and inflammation, worsening the injury. In this report, we describe a patient with a history of a splinter in his left index finger that persisted for three months. The patient was unable to remove the splinter himself, therefore he presented to the clinic for assistance in removal.

## Case presentation

A 62-year-old man presented to the Dermatology clinic for a splinter lodged in his left index volar finger. The object had been present for three months and presented swollen, painful, with increased stratum corneum. He was otherwise in good health with no obvious signs of infection, bleeding, or cellulitis in or around the area. No numbing was required for the procedure. After prepping the skin with alcohol, the physician gently pared away thin layers of stratum corneum with a 15-blade overlying the splinter until the splinter was visualized. Continuing the same paring technique with the 15-blade, the splinter was carefully removed. Complete removal of the splinter was confirmed visually and when there was no further tenderness to palpation.

## Discussion

The classic technique for the removal of a superficial embedded foreign body in the acral skin would be grasping the exposed end of the foreign body with forceps and manual extraction [1]. If a foreign body is positioned horizontally in the skin, extraction may be attempted by making a horizontal incision and extraction with forceps. Deeply embedded vertical foreign bodies can be removed by making an elliptical incision followed by removal with forceps [2,3]. In any of these scenarios, the removal of a foreign body may prove to be painful and challenging. The object requiring removal may be too deeply embedded to allow grasping with forceps. Many painful attempts at removal cause increased swelling and inflammation, exacerbating the difficulty of removal. The increased pain may cause patients to pull back or refuse further attempts, making removal via the physician more difficult. When there is a delay in presentation or failure to promptly remove the foreign body, complications may include pain, swelling, and localized infection [4,5]. To more efficiently remove a small foreign body in the superficial layers of acral skin, there should be a technique that is relatively painless, efficient, and does not require any local anesthetic.

**Figure 1:**
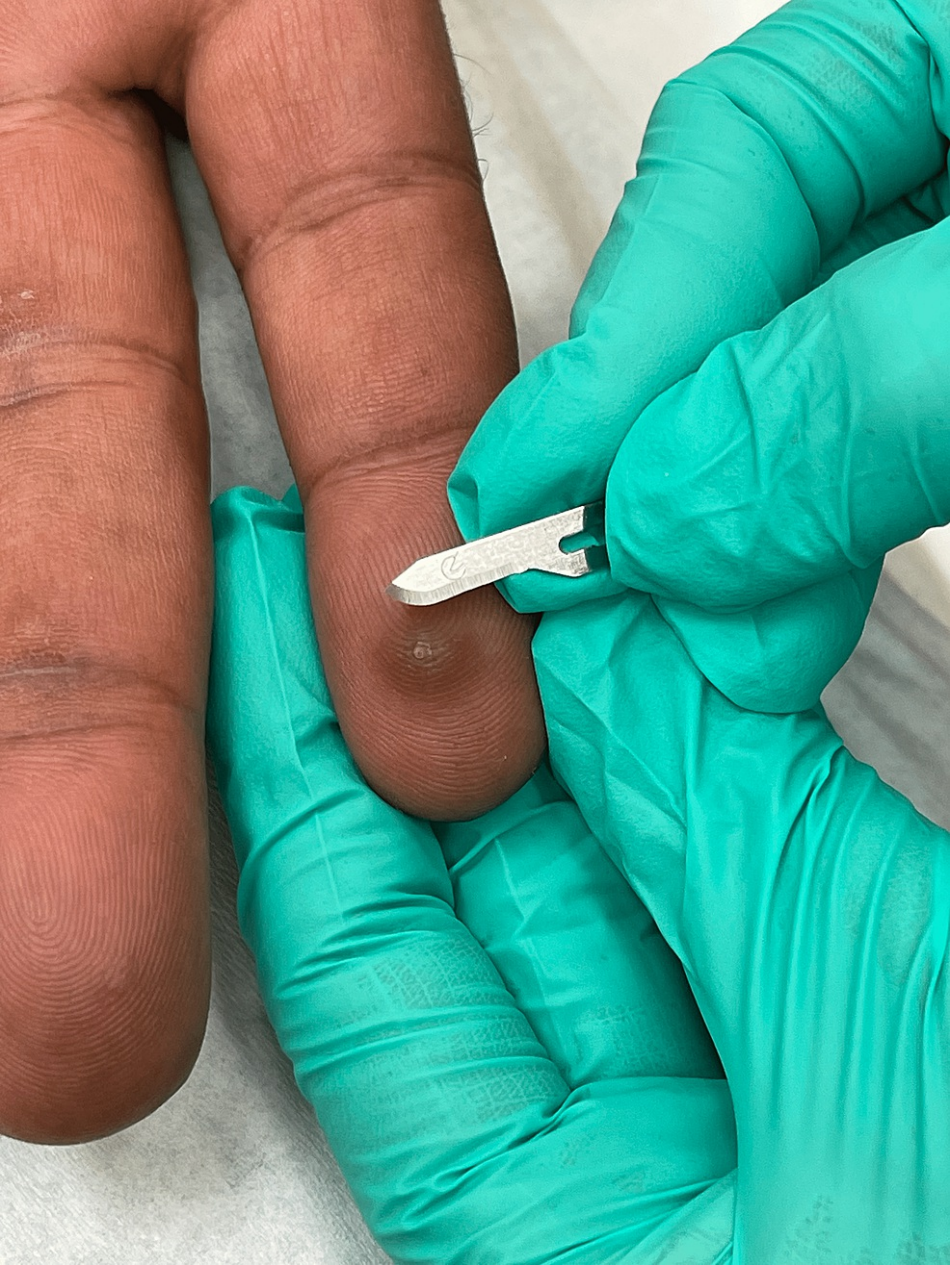
Paring of a Splinter on the Volar Index Finger Using a 15-Blade Scalpel.

**Figure 2:**
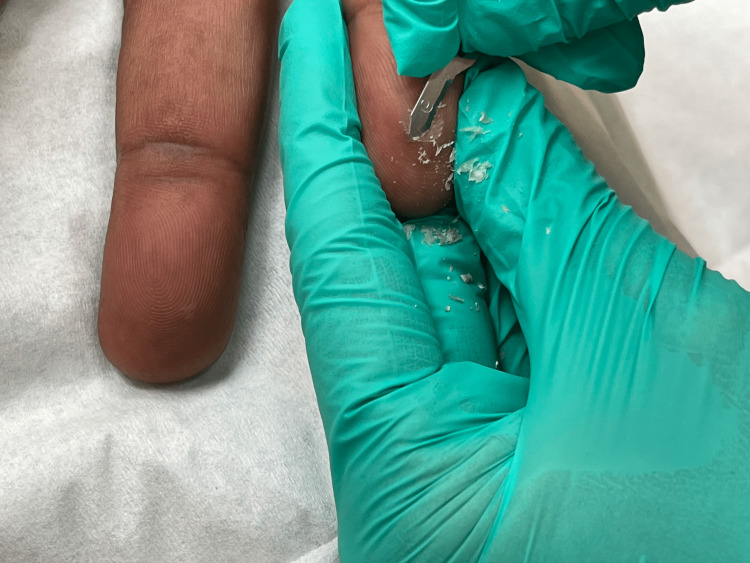
Paring of the Foreign Body Demonstrating Removal of Each Layer of Skin.

Using a paring method, we present a less painful technique to remove a horizontally oriented foreign body presenting in the acral skin. When a patient presents with a foreign body in the acral skin, there is a thick layer of stratum corneum that has no nerve endings. A scalpel, typically a 15-blade, can be utilized for paring of the skin as seen in Figure 1. Each layer is gently and painlessly pared away (Figure 2). Eventually, as layer by layer is removed, the foreign body will present itself. Many times, we see the edge of the object catch the blade and be removed with the next paring of the blade. Other times as the skin is gently pared, the foreign body will become exposed and can be wiped away. The technique is successful when paring longitudinal to the foreign body, following the axis. However, if angled differently, success is still attainable. By focusing on the thin removal of the stratum corneum rather than the foreign body, the patient will have little to no pain and the physician will be more likely to be successful. This procedure works best on acral skin, due to its thick layer of stratum corneum. If the procedure becomes painful and there is a persistent foreign body, the procedure can be terminated, and the patient can return one week later to repeat the procedure and complete foreign body removal. Patients feel little to no pain during this procedure, making them more likely to seek care earlier. Delay in care can cause an increase in pain, inflammation, and risk of infection at the site of the foreign body, making it more difficult for removal later.

## Conclusions

The paring of acral skin with a scalpel blade is a common procedure. This technique can be used to remove a foreign body from any physician’s office. The use of this method reduces pain and complications with foreign body removal and encourages patients to seek professional assistance sooner. This is an efficient, effective, and acceptable method that is simple and less intimidating to patients. There is no numbing or incising involved, reducing stress on the physician and the patient. Patient satisfaction is high with this procedure. Foreign bodies of acral skin are very common, and this method proves to be simple and effective.
